# Iatrogenic Axillary Artery Branch Injury Following Peripherally Inserted Central Catheter (PICC) Line Insertion Managed With Coil Embolization: A Case Report

**DOI:** 10.7759/cureus.84207

**Published:** 2025-05-16

**Authors:** Sohaib Bassam Mahmoud Zoghoul, Saad Ur Rehman, Israa Alhashimi, Rahil H Kassamali, Ali Barah

**Affiliations:** 1 Radiology, Hamad Medical Corporation, Doha, QAT; 2 Medical School, Aston University, Birmingham, GBR

**Keywords:** axillary artery pseudoaneurysm, iatrogenic vascular injury, interventional radiology-guided embolization, microcatheter embolization, picc line complication

## Abstract

Iatrogenic vascular injuries during peripherally inserted central catheter (PICC) placements are uncommon but can lead to significant complications, such as pseudoaneurysms and hematomas. We present the case of a 71-year-old woman with multiple comorbidities who developed a right subpectoral hematoma with active arterial bleeding following PICC line insertion. This injury was successfully managed using minimally invasive coil embolization by the interventional radiology team. This case underscores the importance of early recognition and management of such injuries, highlighting the efficacy of interventional radiology techniques as life-saving interventions.

## Introduction

Peripherally inserted central catheters (PICCs) are widely utilized for long-term venous access in patients with complex medical conditions. Despite their general safety, PICC line placements can occasionally result in complications, including venous perforation and arterial injury [1,2]. The axillary artery, due to its proximity to the axillary vein, is particularly susceptible to iatrogenic injury, which can manifest as pseudoaneurysms or hematomas [1,2]. Early diagnosis and prompt intervention are critical to prevent morbidity and mortality. Minimally invasive techniques, such as transcatheter embolization, offer an effective and less invasive alternative to open surgical repair, especially in critically ill patients [3,4].

## Case presentation

A 71-year-old woman, with a history of coronary artery disease, ischemic cardiomyopathy (ejection fraction of 41%), and recent surgical repair of a posterior gastric wall perforation and type III hiatal hernia, presented with worsening dyspnea, chest pain, and oliguria. She was found to have pneumoperitoneum due to gastric perforation and underwent emergency exploratory laparotomy with repair of the gastric perforation, cruroplasty, gastropexy, and feeding jejunostomy placement.

**Figure 1:**
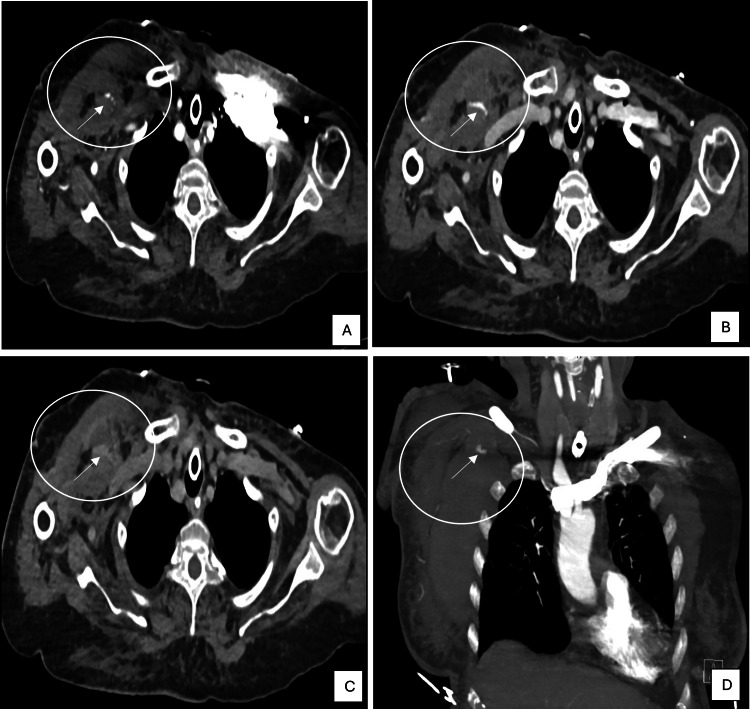
Computed tomography: axial arterial phase (A), axial venous phase (B), axial delayed phase (C), and MIP coronal arterial phase (D) Computed tomography showing the focus of active arterial extravasation progressing on the venous and delayed phases in the vicinity of the right axillary artery (arrows) with large right muscular pectoral/subpectoral hematoma (white circle) MIP: maximum intensity projection

On postoperative day 3, the patient developed right chest swelling around the PICC line insertion site. Computed tomography angiography revealed a large right subpectoral hematoma (8×9×3.7 cm) with active arterial extravasation, suggestive of an axillary artery branch pseudoaneurysm (Figure 1).

The patient's hemoglobin level dropped from 9.7 to 8 g/dL, and she became hemodynamically unstable, necessitating blood product resuscitation. Given the patient's critical condition, open surgical intervention was deemed high risk by the vascular surgery team. The interventional radiology team performed emergency transcatheter embolization under local anesthesia.

**Figure 2:**
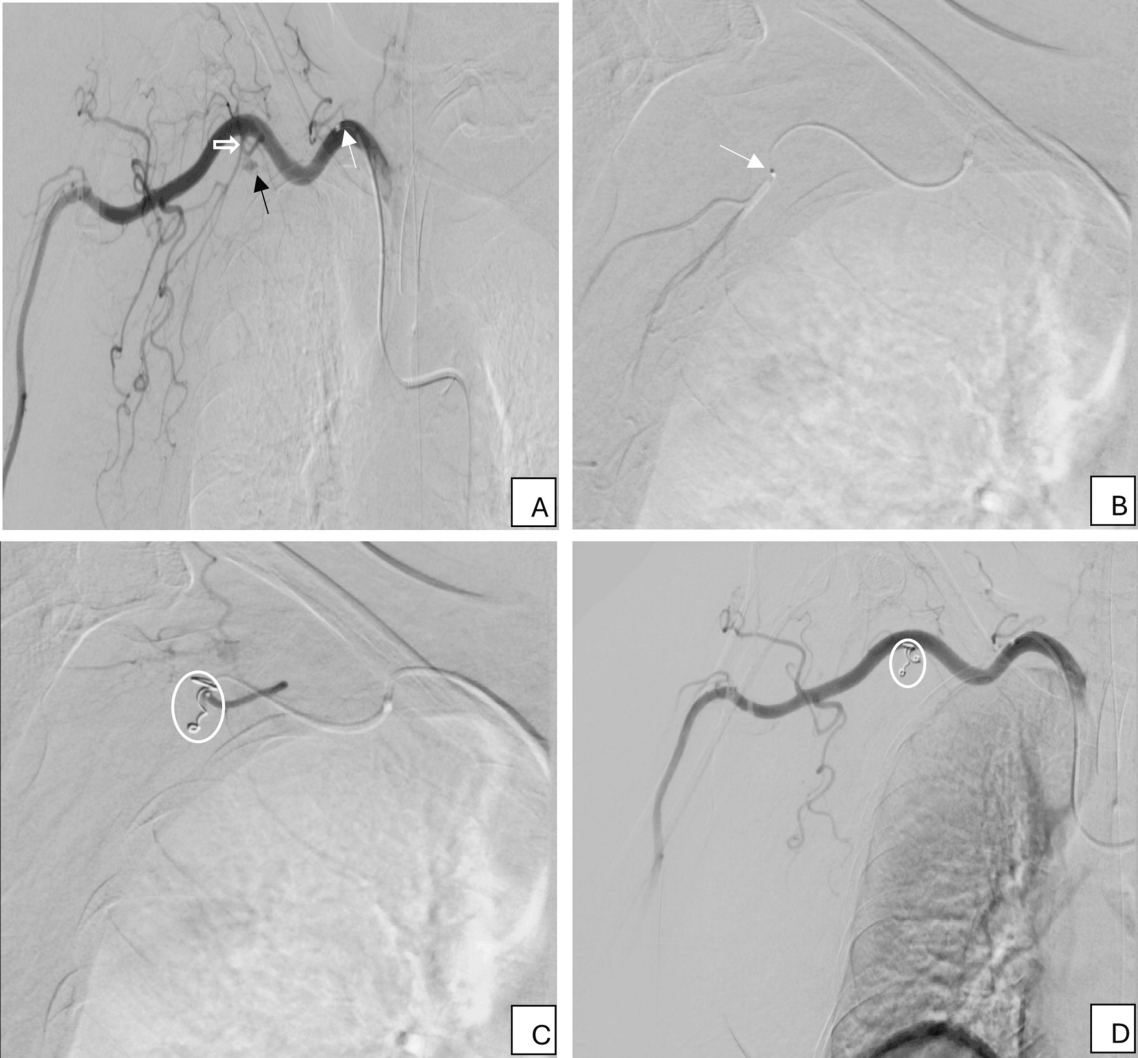
DSA of the right upper limb DSA of the right upper limb with the tip of the angiographic catheter (white thin arrow) in the third part of the subclavian artery showing active arterial blush of extravasation (black thin arrow) from the first part of the axillary artery thoracoacromial branch arising from the common trunk (unfilled white arrow) of the lateral thoracic artery laterally and the thoracoacromial branch superomedially (A). DSA showing the microcatheter system superselectively engaging the common trunk of the lateral thoracic and thoracoacromial branches of the right axillary artery (B). Deployment of Tornado® pushable embolization coils (white circle) in the common trunk of the axillary artery branches (C). Last DSA run showing the successful satisfactory deployment of the coils with the resolution of the active arterial blush (white circle) (D) DSA: digital subtracted angiography

Using a right common femoral artery approach, selective catheterization of the right subclavian artery was achieved with a 5F PIG angiographic catheter (Cordis, Hialeah, Florida, United States). A Direxion™ torqueable microcatheter (Boston Scientific, Marlborough, Massachusetts, United States) was utilized for superselective catheterization of the bleeding axillary artery branch. Embolization was performed with 5 mm×5 cm and 3 mm×2 cm Tornado® embolization microcoils (Cook, Bloomington, Indiana, United States), followed by balloon angioplasty using an 8 mm×4 cm Mustang™ balloon (Boston Scientific, Marlborough, Massachusetts, United States) for stabilization. Final angiography confirmed the complete cessation of bleeding with normal distal flow. Digital subtracted angiography (DSA) of the right upper limb is shown in Figure 2A-2D.

Post-procedure, the patient stabilized hemodynamically, with hemoglobin levels maintained at 9.4 g/dL without further transfusion requirements. No recurrence of the hematoma or pseudoaneurysm was observed on follow-up imaging.

## Discussion

Iatrogenic arterial injuries during PICC line placements are rare but potentially life-threatening complications. The proximity of the axillary vein to the axillary artery increases the risk of arterial injury, particularly in patients with challenging vascular anatomy or coagulopathies. In this case, the inadvertent arterial injury resulted in a pseudoaneurysm and hematoma, necessitating urgent intervention.

Minimally invasive transcatheter embolization is the preferred treatment for iatrogenic arterial injuries in critically ill patients. The use of advanced microcatheters, such as the Direxion™ torqueable microcatheter, facilitates the precise catheterization of target vessels, even in anatomically complex regions [5]. Coil embolization, combined with balloon angioplasty, provides effective hemostasis while preserving distal blood flow.

A review of the literature indicates that successful outcomes have been reported with transcatheter embolization for iatrogenic arterial injuries. Kao and Huang reported a case of iatrogenic axillary artery injury managed successfully with endovascular repair using an 8 mm×5 cm GORE® VIABAHN® stent graft (W. L. Gore & Associates, Newark, Delaware, United States) which was deployed via the right brachial artery for arterial repair, followed by postdilatation with a Rival 8 mm balloon (CR Bard, Murray Hill, New Jersey, United States), and eventually, the pseudoaneurysm was debrided successfully post-procedure [3]. Additionally, the Direxion™ torqueable microcatheter has been highlighted for its advanced torqueability and flexibility, which are beneficial in navigating complex vascular anatomies [4].

This case underscores the importance of early recognition and intervention. Multidisciplinary collaboration between surgery, interventional radiology, and critical care teams is vital for optimizing outcomes. Additionally, routine post-procedural monitoring of PICC line patients is essential for the early detection of complications.

## Conclusions

This case demonstrates the successful management of a rare iatrogenic axillary artery branch injury following PICC line insertion using transcatheter coil embolization. The use of advanced microcatheter systems and embolization techniques enabled effective hemostasis in a critically ill patient, avoiding the risks associated with open surgery. This case highlights the critical role of interventional radiology in managing complex vascular injuries and emphasizes the need for vigilance during and after PICC line placements.
